# Vaccine Platform-Dependent Differential Impact on Microbiome Diversity: Potential Advantages of Protein Subunit Vaccines

**DOI:** 10.3390/vaccines13121248

**Published:** 2025-12-15

**Authors:** Hye Seong, Jin Gu Yoon, Eliel Nham, Yu Jung Choi, Ji Yun Noh, Hee Jin Cheong, Woo Joo Kim, Sooyeon Lim, Joon Young Song

**Affiliations:** 1 Department of Internal Medicine, Yonsei University College of Medicine, Seoul 03722, Republic of Korea; shininghye@yuhs.ac; 2 Department of Internal Medicine, Korea University College of Medicine, Seoul 08308, Republic of Korea; kormid@korea.ac.kr (J.G.Y.); e.nham@kumc.or.kr (E.N.); pm94@naver.com (Y.J.C.); jynoh@korea.ac.kr (J.Y.N.);; 3 Vaccine Innovation Center-KU Medicine, Seoul 08308, Republic of Korea; 4 Asia Pacific Influenza Institute, Korea University College of Medicine, Seoul 08308, Republic of Korea

**Keywords:** SARS-CoV-2, protein subunit vaccine, gut microbiome, vaccine immunogenicity, booster vaccination

## Abstract

**Background**: The COVID-19 pandemic accelerated the development of diverse vaccine platforms, including mRNA, adenoviral vector, and protein subunit vaccines. Given the growing evidence that the gut microbiome modulates vaccine-induced immunity, this study compared the effects of a protein subunit vaccine (NVX-CoV2373), an mRNA vaccine (BNT162b2), and an adenoviral vector vaccine (ChAdOx1) on gut microbiome diversity following booster vaccination. **Methods**: We conducted a prospective cohort study involving 35 healthy adults who received an NVX-CoV2373 booster. Stool and blood samples were collected before vaccination and three weeks afterward. Gut microbiome profiles were analyzed using 16S rRNA gene sequencing, and the results were compared with our previous cohorts who received BNT162b2 or ChAdOx1 vaccines. **Results**: The NVX-CoV2373 booster was associated with a significant increase in the Shannon diversity index (*p* = 0.027), indicating enhanced alpha diversity. This finding contrasts with the decrease or absence of significant short-term change observed following repeated administrations of adenoviral vector and mRNA vaccines, respectively. Notably, NVX-CoV2373 vaccination was accompanied by an increased relative abundance of beneficial taxa such as *Bacteroides fragilis* and a decrease in *Prevotella bivia*. In comparison, repeated ChAdOx1 doses resulted in a sustained reduction in alpha diversity, whereas BNT162b2 showed a transient post-booster rise followed by a long-term decline in species richness. **Conclusions**: In the booster setting, the protein subunit vaccine NVX-CoV2373 exerted a distinct and favorable effect on gut microbiome diversity, increasing alpha diversity in contrast to the patterns observed with mRNA and adenoviral vector booster vaccines.

## 1. Introduction

The coronavirus disease 2019 (COVID-19) pandemic accelerated the rapid development of diverse vaccine platforms based on distinct immunological mechanisms, including messenger RNA (mRNA), adenoviral vector, and protein subunit technologies. Among them, mRNA vaccines such as BNT162b2 (Comirnaty™) and mRNA-1273 (Spikevax™) deliver lipid nanoparticle-encapsulated mRNA, enabling host cells to transiently express these antigens and elicit robust immune responses. Adenoviral vector vaccines, represented by ChAdOx1 (Vaxzevria™), utilize replication-deficient viral carriers to deliver SARS-CoV-2 genetic material, thereby inducing antigen-specific immunity. Protein subunit vaccines, as the most recently introduced platform, including NVX-CoV2373 (Nuvaxovid™) and GBP510 (Skycovione™), consist of purified viral proteins formulated with adjuvants to stimulate targeted immune activation [1,2,3].

The gut microbiome has emerged as an important modulator of host immunity, influencing both baseline immune function and vaccine responsiveness [4,5,6]. A balanced and diverse microbiome is often associated with enhanced immune function, whereas dysbiosis, defined as an imbalance in the microbial community, can impair vaccine efficacy and promote inflammation [7]. Our previous research revealed a distinct relationship between vaccine type and gut microbiome dynamics following COVID-19 vaccination [8]. Specifically, repeated administration of adenoviral vector vaccines led to a significant reduction in microbiome alpha diversity, whereas mRNA vaccines maintained microbial stability [8]. These findings suggest that different vaccine platforms may exert differential effects on host–microbiome interactions and, consequently, on immune outcomes.

As SARS-CoV-2 continues to evolve and vaccine-induced immunity wanes, repeated booster immunizations have become an integral component of global vaccination strategies. However, certain vaccine platforms may induce dysbiosis, which in turn could compromise the effectiveness of subsequent doses. Therefore, elucidating the long-term impact of different vaccine technologies on the gut microbiome is essential for optimizing both the efficacy and safety of repeated immunization.

Protein subunit vaccines may modulate the gut microbiome differently from mRNA and adenoviral vector vaccines because they rely on exogenously delivered antigens and adjuvant-mediated innate immune activation [9]. NVX-CoV2373 contains the Matrix-M saponin adjuvant, which enhances antigen-presenting cell activation and cytokine production through extracellular pattern-recognition pathways, in contrast to the intracellular immune signaling induced by mRNA and adenoviral vector platforms [10]. These platform-specific differences in early innate immune responses may lead to distinct downstream effects on gut microbial composition.

In this context, we hypothesized that the NVX-CoV2373 booster would increase or preserve gut microbiome alpha diversity and induce distinct taxonomic and functional microbial shifts compared with mRNA and adenoviral vector vaccines. To test this hypothesis, we examined gut microbiome changes following booster immunization with the protein subunit vaccine NVX-CoV2373 and compared these findings with microbiome responses to the mRNA vaccine BNT162b2. Building on our previous work, we aimed to elucidate the platform-dependent differential impacts of protein subunit, adenoviral vector, and mRNA vaccines on gut microbial diversity and community composition.

## 2. Methods

### 2.1. Experimental Model and Participant Details

#### 2.1.1. NVX-CoV2373 Booster Cohort

Between 19 April and 6 May 2022, we prospectively recruited 35 healthy adults at a tertiary hospital in Seoul, Republic of Korea, who received the NVX-CoV2373 vaccine as a booster dose. Individuals who had taken medications that could affect the gut microbiota (e.g., antibiotics, laxatives, or motility agents) within one month prior to vaccination or who had a history of SARS-CoV-2 infection were excluded. In the Republic of Korea, NVX-CoV2373 was introduced exclusively for booster administration rather than for primary vaccination. Accordingly, all NVX-CoV2373 recipients in this study had already completed their primary series with BNT162b2 or ChAdOx1 prior to enrollment, and baseline sampling was conducted at the pre-booster visit (B1). Therefore, V1–V3 sampling schedule, which was applicable to the primary-series cohorts, was not applicable to the NVX-CoV2373 group.

Serial fecal and blood samples were collected at two time points: immediately before booster administration (B1) and three weeks afterward (B2). Participant information, including demographics, medication history, use of probiotics and supplements, laboratory findings, and dietary records, was systematically collected by two trained physicians to ensure consistency.

The study was approved by the Institutional Review Board of Korea University Guro Hospital (IRB No. 2021GR0099). All participants provided written informed consent, and all procedures adhered to the Declaration of Helsinki (1964) [11] and its subsequent amendments.

#### 2.1.2. BNT162b2 Booster Cohort

Following the methodology described in our previous study [8], we recruited 53 healthy healthcare workers at the same tertiary hospital from 25 February to 16 July 2021. These participants were assigned to receive either BNT162b2 (n = 27) or ChAdOx1 (Oxford/AstraZeneca) (n = 26) vaccines. We initially analyzed microbiome changes at three time points: before the first dose (V1), before the second dose (V2), and three weeks after the second dose (V3). In this extension of the study, we focused on participants who received the BNT162b2 booster six months after the two-dose primary series and assessed microbiome changes before (V4) and three weeks after (V5) the BNT162b2 booster. Exclusion criteria were identical to the NVX-CoV2373 cohort.

Serial fecal and blood samples were collected at each time point. Two trained physicians systematically collected clinical and lifestyle data to ensure accuracy and consistency.

The Institutional Review Board of Korea University Guro Hospital approved this study protocol (2021GR0097). We obtained written informed consent from all participants and adhered to the ethical standards of the relevant institutional and national research committees by the Declaration of Helsinki of 1964 and its subsequent amendments.

### 2.2. Sample Collection and Processing

Fecal samples were collected using DNA Preservation & Transport Kits for Fecal Swabs (Noble Bio, Hwaseong, Republic of Korea), which contain a medium for nucleic acid preservation. These samples, once placed in the fecal swab transport medium, were maintained at a temperature of −80 °C. Blood samples were obtained by venipuncture into serum separation tubes and centrifuged at 2500 rpm for 10 min at 4 °C. Serum was aspirated into sterile screw-cap vials and stored at −80 °C.

### 2.3. Immunoassay for Quantitative Determination of Antibodies Against the SARS-CoV-2 Spike Protein

Blood samples were analyzed to assess humoral immune responses. The Elecsys^®^ Anti-SARS-CoV-2 S assay kit from Roche, Rotkreuz, Switzerland, was used to determine anti-S levels according to the manufacturer’s guidelines. Titers below the limit of quantitation were assigned a value of 0.4 U/mL.

Participants who received the NVX-CoV2373 booster vaccine were categorized into high- and low-responder groups based on their fold increase in anti–SARS-CoV-2 spike (S) IgG titers from baseline (B1) to three weeks post-vaccination (B2). Individuals showing a ≥3-fold increase were defined as high responders, whereas those showing a ≤2-fold increase were classified as low responders.

### 2.4. Microbiological Analysis

#### 2.4.1. DNA Extraction, PCR Amplification, and Sequencing

Total DNA was extracted from the samples using the FastDNA^®^ SPIN Kit for Soil (MP Biomedicals, Santa Ana, CA, USA) according to the manufacturer’s protocol. The V3-V4 regions of the 16S rRNA gene were targeted for PCR amplification using the extracted DNA as a template. The fusion primers used for bacterial amplification were 341F (5’-AATGATACGGCGACCACCGAGATCTACAC-XXXXXXXXTCGTCGGCAGCGTC-AGATGTGTATAAGAGACAG-CCTACGGGNGGCWGCAG-3’, where the underlined sequence is the target region primer) and 805R (5’-CAAGCAGAAGACGGCATACGAGAT-XXXXXXXXGTCTCGTGGGCTCGG-AGATGTGTATAAGAGACAG-GACTACHVGGTATCTAATCC-3’). The primers were designed as follows: P5 (P7) adapter, i5 (i7) index, Nextera consensus sequence, sequencing adapter, and target region sequence. PCR conditions consisted of an initial denaturation at 95 °C for 3 min, followed by 25 cycles of denaturation at 95 °C for 30 s, 55 °C for 30 s, and 72 °C for 30 s, with a final extension at 72 °C for 5 min. PCR products were verified on a 1% agarose gel and visualized using a Gel Doc system (Bio-Rad, Hercules, CA, USA). PCR products were then purified using the CleanPCR kit (CleanNA, Waddinxveen, The Netherlands), pooled at equal concentrations, and short non-target fragments were removed using the same kit. The quality and size of the products were assessed using a Bioanalyzer 2100 system (Agilent, Palo Alto, CA, USA) with a DNA 7500 chip. The pooled amplicons were sequenced at CJ Bioscience, Inc. (Seoul, Republic of Korea) on the Illumina MiSeq Sequencing System (Illumina, San Diego, CA, USA) according to the manufacturer’s instructions.

#### 2.4.2. DNA Analysis Pipeline

The initial quality check of the raw reads was performed using Trimmomatic ver. 0.32, which filtered out reads with a quality score below Q25. After quality control, paired-end sequences were merged using the fastq_mergepairs command in VSEARCH ver.2.13.4 using default parameters. Primer trimming was performed using the Myers–Miller alignment algorithm [12] with a similarity threshold of 0.8. Non-specific amplicons, such as those not encoding 16S rRNA, were identified using the nhmmer algorithm [13] in the HMMER software package ver.3.2.1 with hmm profiles. Unique reads were extracted, and redundant reads were clustered with these unique sequences using the deep-full-length command in VSEARCH [14]. Taxonomic assignment was performed using the EzBioCloud 16S rRNA database [15] using VSEARCH’s usearch_global command [14], followed by precise pairwise alignment [12]. Chimeric sequences were identified and removed using the UCHIME algorithm [16] and the EzBioCloud non-chimeric 16S rRNA database, excluding reads with <97% sequence similarity. Reads not classified to the species level in the EzBioCloud database were pooled and de novo clustering was performed using the cluster_fast command [14] to generate additional operational taxonomic units (OTUs). Single-read OTUs were excluded from further analyses. Subsequent analyses, including diversity metrics and biomarker identification, were performed using the EzBioCloud 16S-based MTP, a bioinformatics cloud platform developed by CJ Bioscience, Inc. (Seoul, Republic of Korea; https://www.ezbiocloud.net/, accessed on 8 December 2025).

The alpha diversity indices (ACE [17], Chao1 [18], Jackknife [19], Shannon [20], NPShannon [21], Simpson [20], and phylogenetic diversity [22]) were calculated as described in previous studies. Beta diversity distances were determined using the Jensen–Shannon [23], Bray–Curtis [24], Generalized UniFrac [25], and Fast UniFrac [26] algorithms. Functional profiles were predicted using PICRUSt [27], and MinPath [28], and taxonomic and functional biomarkers were identified using linear discriminant analysis effect size (LEfSe) [29]. Prior to analysis, all microbiome count data were normalized to 1000 reads. All analyses were conducted on the EzBioCloud 16S-based MTP, the bioinformatics cloud platform developed by CJ Bioscience, Inc. (https://www.ezbiocloud.net, accessed on 8 December 2025).

### 2.5. Statistical Analysis

All continuous variables in the study are presented as medians, with interquartile ranges (IQRs) calculated as the difference between the third and first quartiles. Non-parametric statistical methods were used to assess differences between groups. Categorical variables are expressed as counts and percentages. The Mann–Whitney U test was used to compare the low- and high-responder groups. In addition, paired *t*-test and Wilcoxon signed-rank test were used for within-group comparisons between the pre- and post-vaccination groups. All statistical analyses were performed using R statistical software, version 4.1.1 (R Foundation for Statistical Computing, Vienna, Austria).

## 3. Results

### 3.1. NVX-CoV2373 Booster Increases Gut Microbiome Alpha Diversity

**Figure 1:**
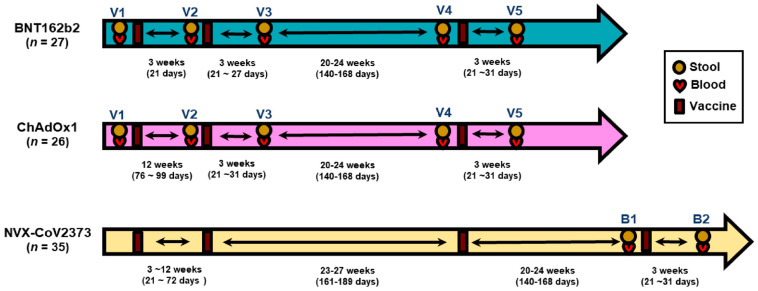
**Study design and sample collection timeline.** Schematic overview of study protocols for the three vaccine cohorts. Participants receiving BNT162b2 (n = 27) and ChAdOx1 (n = 26) vaccines were followed longitudinally at five time points (V1–V5): before the first dose (V1), before the second dose (V2), three weeks after the second dose (V3), approximately 20–24 weeks after the second dose (V4), and three weeks after booster vaccination (V5). The NVX-CoV2373 cohort (n = 35) was assessed at two time points: before (B1) and three weeks after (B2) booster administration. Symbols indicate sample collection points for stool (●), blood (♥), and vaccine administration (▮). Arrows represent time intervals between visits.

**Figure 2:**
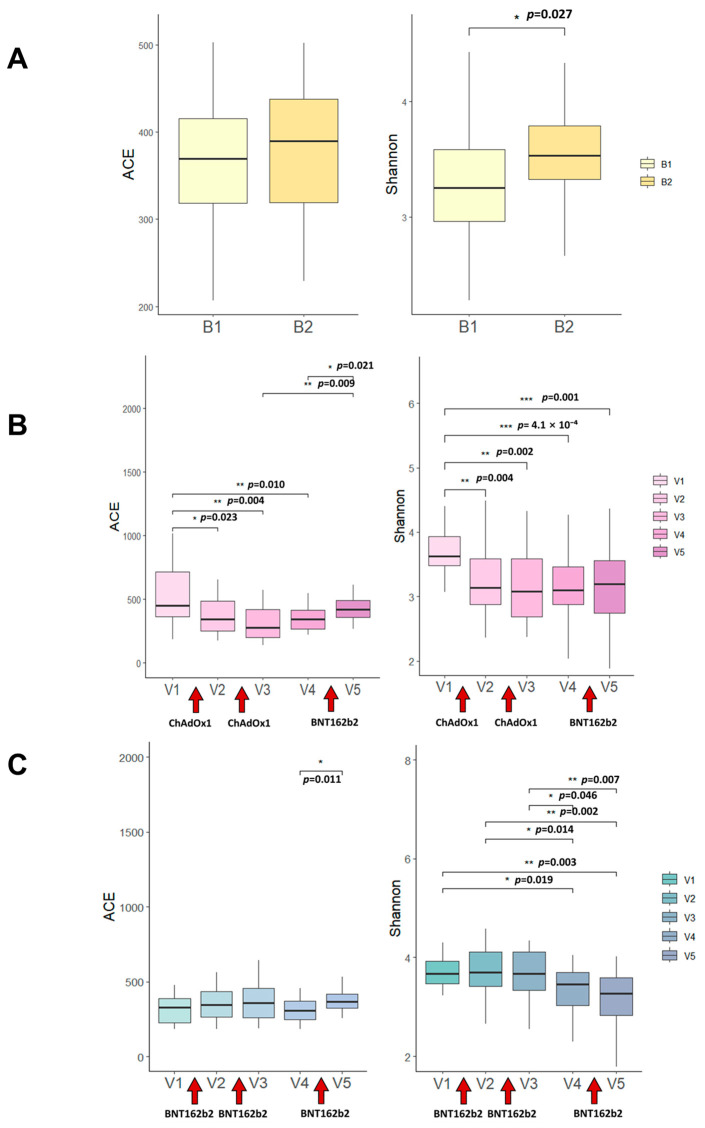
**Changes in gut microbiome alpha diversity following COVID-19 vaccination.** (**A**) ACE and Shannon diversity indices of the gut microbiome before (B1) and three weeks after (B2) NVX-CoV2373 booster administration. The Shannon index significantly increased after vaccination (*p* = 0.027; Wilcoxon signed-rank test). (**B**) Longitudinal changes in alpha diversity (ACE and Shannon indices, Cohen’s d = 0.45, CI: −0.45 to 0.006) among individuals receiving the ChAdOx1 vaccine. Diversity decreased after the first and second doses and remained low up to 5–6 months post-vaccination. (**C**) Longitudinal changes in alpha diversity (ACE and Shannon indices) in individuals receiving the BNT162b2 vaccine. Diversity was relatively stable following the primary doses but decreased 5–6 months after dose 2 and increased after the BNT162b2 booster. Boxplots show medians and interquartile ranges. Asterisks or *p*-values indicate statistically significant differences between time points(*p* < 0.05, *, *p* < 0.01, **, *p* < 0.001, ***).

We analyzed the gut microbiome of 35 healthy adults before (B1) and three weeks after (B2) administration of the NVX-CoV2373 booster vaccine (Figure 1). Baseline characteristics and antibody responses of the participants are summarized in Table 1. Microbiome analysis showed a significant increase in the Shannon diversity index after the NVX-CoV2373 booster (*p* = 0.027), indicating enhanced alpha diversity (Figure 2A). In contrast, principal coordinate analysis (PCoA) based on Bray–Curtis distances revealed no significant difference in overall microbial community composition (beta diversity) between the B1 and B2 time points.

### 3.2. Taxonomic and Functional Shifts Following NVX-CoV2373 Booster

Differences in relative taxonomic abundance (LDA effect size > 2.5) between pre- and post-booster samples are shown in Table 2. Notably, the relative abundance of *Prevotella bivia* decreased after booster administration, whereas *Bacteroides fragilis*, *Fusobacterium necrogenes*, *Akkermansia muciniphila*, *Ruminococcaceae* PAC000661_g PAC001052_s, *Collinsella aerofaciens*, and *Oscillibacter* KI271778_s increased. Species-level differences identified by paired *t*-tests (Table 3) were consistent with the LDA results, showing significant post-vaccination increases in *B. fragilis* and *Oscillibacter* KI271778_s.

Functional predictions based on taxonomic profiles (Table 4) revealed distinct metabolic alterations following booster vaccination. PICRUSt-based pathway analysis indicated significant enrichment of module M00207 (putative multiple sugar transport system) and module M00038 (tryptophan metabolism; tryptophan → kynurenine → 2-aminomuconate), accompanied by a reduction in module M00126 (tetrahydrofolate biosynthesis; GTP → THF).

### 3.3. Comparative Analysis with mRNA and Adenovirus Vector Vaccines

In our previous study, we analyzed gut microbiome changes at three time points: prior to the first dose (V1), prior to the second dose (V2), and three weeks after the second dose (V3) in individuals who received two doses of either BNT162b2 or ChAdOx1 (Figure 1). In the present study, the observation period was extended to six months after completion of the two-dose primary series. At this point, participants received a BNT162b2 booster dose (V4), and microbiome profiles were evaluated both before (V4) and three weeks after (V5) booster administration.

Individuals who had initially received ChAdOx1 exhibited a significant reduction in alpha diversity after both the first and second doses, which persisted for up to 5–6 months following the second dose (Figure 2B). In contrast, recipients of BNT162b2 showed no short-term changes in alpha diversity, although a significant decline was observed 5–6 months after the second dose compared with baseline (Figure 2C). Notably, administration of the BNT162b2 booster significantly increased species richness in both BNT162b2- and ChAdOx1-primed individuals, without a corresponding change in overall diversity indices.

By contrast, the NVX-CoV2373 protein subunit vaccine elicited a significant short-term increase in gut microbiome alpha diversity (Figure 2A), whereas the other vaccine platforms were generally associated with a reduction in diversity following repeated vaccination.

### 3.4. Microbiome Diversity and Humoral Immune Response

**Figure 3:**
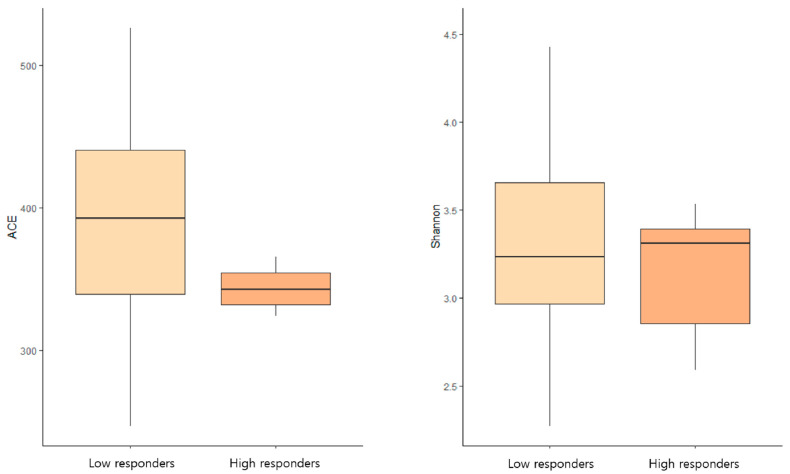
**Comparison of gut microbiome alpha diversity between high and low NVX-CoV2373 responders.** Boxplots show ACE and Shannon diversity indices in high responders (≥3-fold increase in anti-SARS-CoV-2 S IgG titers from B1 to B2) and low responders (≤2-fold increase). No significant differences were observed between groups (*p* = 0.694; Wilcoxon rank-sum test). Boxes represent interquartile ranges, horizontal lines indicate medians, and whiskers denote minimum and maximum values.

We investigated the association between gut microbiome composition and the humoral immune response to the NVX-CoV2373 booster vaccine. No significant differences were observed in either alpha or beta diversity of the gut microbiota between high- and low-responder groups (*p* = 0.694; Figure 3). Table 5, Table 6 and Table 7 summarize detailed comparisons of participant characteristics, taxonomic biomarkers, and functional modules between high and low responders following NVX-CoV2373 booster vaccination. Although alpha diversity did not differ significantly between high and low responders, the ACE richness index in low responders clustered within a comparatively uniform lower range, suggesting a tendency toward reduced baseline richness in this subgroup (Table 5). Baseline taxonomic profiling identified multiple genera and species with differential abundance between responders (Table 6). High responders were enriched in *Ruminococcus_g5, Haemophilus, Romboutsia, Agathobaculum*, and *Faecalimonas*, whereas low responders showed higher levels of *Alloprevotella, Agathobacter*, and *Paraprevotella*. Functional profiling (Table 7) revealed only modest differences in predicted metabolic pathways, with no single immune-related module distinguishing the responder groups. This suggests that baseline taxonomic composition, rather than predicted functional capacity, may be a more sensitive indicator of variation in humoral responsiveness.

## 4. Discussion

This study demonstrates that the protein subunit vaccine NVX-CoV2373 exerts a distinct effect on gut microbiome composition compared with mRNA and adenoviral vector vaccines. Unlike the reductions in microbial alpha diversity typically observed following repeated administration of other vaccine platforms, NVX-CoV2373 was associated with a significant short-term increase in microbiome diversity. These findings suggest that protein subunit vaccines may help restore and stabilize gut microbial balance during booster immunization.

Interpretation of alpha-diversity changes requires consideration of the distinct ecological dimensions captured by different indices. The Shannon index reflects both richness and evenness, whereas the ACE index emphasizes rare-species richness; therefore, it is not uncommon for these metrics to differ in statistical significance within the same dataset. In this study, our conclusion that NVX-CoV2373 elicited a more favorable short-term microbiome response is based on the overall direction and coherence of the observed shifts rather than on any single index. NVX-CoV2373 produced concordant increases in both Shannon and ACE values, indicating simultaneous gains in richness and evenness, with Shannon reaching statistical significance. In contrast, the BNT162b2 booster yielded a discordant pattern, with ACE increasing but Shannon declining, a result that is consistent with an expansion of rare taxa without improvement in community evenness. Moreover, NVX-CoV2373 was uniquely associated with enrichment of beneficial bacterial species. Taken together, these taxonomic and diversity-based signatures support the interpretation that NVX-CoV2373 induces a more balanced and potentially favorable microbiome shift during booster vaccination, even when individual diversity indices differ in statistical significance because of their inherent ecological properties. Specifically, the enrichment of *Bacteroides fragilis* and *Oscillibacter* species observed after NVX-CoV2373 vaccination may provide important insights into host–microbiome interactions. To better contextualize these findings, differentially abundant taxa were further grouped according to their functional and metabolic characteristics. Increases in *Akkermansia muciniphila*, *Bacteroides fragilis*, and members of Ruminococcaceae family—taxa associated with mucin degradation, short-chain fatty acid (SCFA) production, and immune regulation—suggest a shift toward a more metabolically favorable and anti-inflammatory gut environment following NVX-CoV2373 boosting. Conversely, the reduction in *Prevotella bivia*, a species linked to inflammatory mucosal states, may also contribute to improved microbial homeostasis. *B. fragilis* exhibits immunomodulatory properties and has the capacity to correct gut dysbiosis, thereby promoting immune homeostasis and enhancing host defense [30]. Although data on *Oscillibacter KI271778_s* remain limited, members of the Ruminococcaceae family are known to participate in secondary bile acid production, which can attenuate intestinal inflammation and support immune regulation [31].

Furthermore, functional pathway analysis revealed upregulation of modules associated with multiple sugar transport and tryptophan metabolism, both of which are intricately linked to gut homeostasis and immune regulation. Enhanced microbial carbohydrate transport facilitates nutrient exchange and the fermentation of dietary fibers into SCFAs such as acetate, propionate, and butyrate [32,33,34]. SCFAs play essential roles in maintaining intestinal barrier integrity by lowering luminal pH, promoting mucus production, and providing energy to epithelial cells [33]. In addition, they modulate host immune responses, contributing to mucosal immune tolerance and suppression of inflammation [32,35]. Collectively, these processes support gut homeostasis, and are linked to improved metabolic health and a reduced risk of gastrointestinal and metabolic disorders [33,34].

Alterations in tryptophan metabolism may influence mucosal immunity through increased production of indole derivatives that activate aryl hydrocarbon receptor (AHR), which enhances epithelial barrier function and regulates IL-22–dependent mucosal responses [36,37]. Consistent with this pathway, tryptophan-derived microbial metabolites, such as indole-3-propionate and indole-3-lactate, act as signaling molecules that reinforce intestinal barrier integrity, promote epithelial renewal, and modulate mucosal immune responses [38,39,40]. Moreover, these metabolites facilitate the differentiation of anti-inflammatory regulatory T cells and macrophages through activation of the AHR pathway, a key regulator of mucosal immunity and intestinal tolerance [41,42]. Conversely, the predicted reduction in tetrahydrofolate (THF) biosynthesis likely reflects community-level adjustments in folate-dependent one-carbon metabolic capacity rather than a taxon-specific change, consistent with the broader metabolic shifts observed after NVX-CoV2373 boosting [43]. Collectively, these metabolic and compositional changes indicate a gut microbiome milieu that supports mucosal immune resilience and may enhance the durability of vaccine-induced protection. Overall, these functional and taxonomic signatures suggest that the protein subunit vaccine platform can beneficially modulate the intestinal ecosystem to promote balanced and effective immune activation.

Several mechanisms may underlie the favorable microbiome profile observed after NVX-CoV2373 vaccination. The Matrix-M saponin adjuvant may indirectly support microbial homeostasis by enhancing balanced innate immune activation and promoting regulatory immune signaling rather than excessive inflammation [10,44]. Systemic immune activation following vaccination could also reinforce mucosal immune tone and epithelial barrier integrity, maintaining a stable nutrient and metabolic environment that favors microbial diversity. Although these mechanisms remain speculative, they provide a plausible biological framework linking the immunological characteristics of protein subunit vaccines to the preservation of gut microbial balance and diversity observed in this study.

Overall alpha diversity was comparable between responder groups, but baseline microbial signatures are more closely associated with humoral responsiveness. The taxa enriched in high versus low responders suggest differences in community function, including SCFA production and inflammatory modulation, which may shape vaccine-induced antibody responses. These patterns align with previous evidence demonstrating that gut microbiota composition influences BNT162b2 immunogenicity, reinforcing the concept that the microbiome can modulate host vaccine responsiveness. Importantly, our findings provide the first indication that similar microbiome–immunity relationships may also affect responses to the NVX-CoV2373 protein subunit vaccine. This suggests that inter-individual variability in baseline microbial ecosystems may contribute to the heterogeneity of booster vaccine responses, even when the vaccine platform itself exhibits a favorable effect on microbial diversity.

The gut microbiome plays a pivotal role in determining baseline immune status and shaping vaccine-induced immune responses [4,5,6]. Previous studies have shown that reduced microbial diversity and abundance correlate with heightened inflammation and diminished vaccine responses, as demonstrated in a human microbiome intervention study using a trivalent influenza vaccine [7]. Consistent with our earlier findings that pre-vaccination dysbiosis was linked to weaker antibody responses after COVID-19 vaccination [8], the maintenance or enhancement of microbiome diversity observed following NVX-CoV2373 administration may confer an advantage in sustaining immune competence during repeated immunization cycles. Protein subunit vaccines generally elicit moderate immune responses after the primary dose, with more pronounced immunogenicity observed upon homologous boosting [1,2]. This enhanced response pattern may reflect the favorable microbiome alterations identified in this study.

This study has several limitations. First, only a single booster dose of NVX-CoV2373 was evaluated, and long-term microbiome trajectories beyond the three-week post-vaccination period were not assessed. Second, functional inferences were derived from 16S rRNA gene sequencing–based predictive metagenomic profiling rather than shotgun metagenomics or direct metabolomic measurements, which may limit the resolution and accuracy of pathway-level interpretations. Third, although dietary intake was not experimentally controlled during follow-up, all participants completed validated food-frequency questionnaires, and no meaningful changes in dietary pattern or nutrient intake were observed during the study period, reducing the likelihood that diet-related fluctuations confounded the findings. Fourth, cross-cohort comparisons should be interpreted cautiously, as the NVX-CoV2373, BNT162b2, and ChAdOx1 cohorts were enrolled during different time periods and involved populations with varying demographic and environmental characteristics. Although our analyses focused on within-individual longitudinal changes to minimize inter-individual variability, unmeasured factors—such as age differences, seasonal timing, lifestyle variation, and background SARS-CoV-2 prevalence—may still have contributed to subtle differences in microbiome outcomes. Finally, the responder subgroup analysis included a modest sample size, which may limit statistical power to detect more subtle microbiome–immunogenicity associations. Nevertheless, all participants across cohorts were generally healthy adults, with only a few individuals having well-controlled hypertension or diabetes mellitus. Combined with the shared ethnic background and the absence of substantial dietary differences between cohorts, these characteristics partially mitigate concerns regarding cohort heterogeneity. Future longitudinal, multi-omic studies integrating metagenomics, metabolomics, and immune profiling will be needed to elucidate the causal relationships between microbiome alterations and vaccine-induced immunogenicity.

Overall, these findings highlight the potential of the NVX-CoV2373 protein subunit vaccine to modulate the gut microbiome in a manner distinct from mRNA and adenoviral vector platforms. A deeper understanding of host–microbiome–vaccine interactions may guide the development of personalized, precision vaccination strategies tailored to individual immune-microbiome profiles, particularly in the context of repeated immunization against SARS-CoV-2, influenza, and respiratory syncytial virus.

## 5. Conclusions

In summary, this study provides evidence that the protein subunit vaccine NVX-CoV2373 exerts a distinct effect on the gut microbiome compared with mRNA and adenoviral vector vaccines. Unlike other vaccine platforms that tend to reduce microbial alpha diversity following repeated administration, NVX-CoV2373 was associated with a significant short-term increase in microbiome diversity and the enrichment of beneficial taxa such as *Bacteroides fragilis* and *Oscillibacter* species. Functional pathway enrichment, particularly in tryptophan metabolism and carbohydrate transport, supports the notion that this vaccine platform may promote a metabolically and immunologically favorable intestinal environment.

These findings suggest that NVX-CoV2373 may help maintain gut microbial balance during booster vaccinations, thus aiding immune response and reducing dysbiosis. Further longitudinal and mechanistic studies are warranted to clarify the causal relationships between vaccine-induced microbiome modulation and host immune outcomes. Understanding these interactions will be critical for optimizing vaccination strategies and enhancing the safety and efficacy of current and emerging vaccine platforms.

Building on these insights, our findings raise practical considerations for future booster strategies. Incorporating microbiome assessments into vaccine studies may clarify how host–microbiome interactions shape vaccine-induced immunity, particularly among older adults or individuals with preexisting dysbiosis who may mount suboptimal responses. As the microbiome is increasingly recognized as a modifiable determinant of vaccine effectiveness, systematic microbiome monitoring in clinical vaccine research could inform the design of booster strategies that optimize immunogenicity and support more individualized vaccination approaches.

## Figures and Tables

**Table 1:** Baseline characteristics and antibody responses of participants.

Characteristics	B1 (*n* = 35)	B2 (*n* = 35)	*p* Value
**Age (years)**	65 ± 4.2		
**Female sex (%)**	23 (65.7)		
**BMI (kg/m^2^)**	23.7 ± 2.5		
**Anti-SARS-CoV-2 S IgG (U/mL)**	12,146.1 ± 11,573.2	15,156.5 ± 13,015.8	<0.001
**Laboratory test results**			
WBC (10^3^/µL)	5.5 ± 1.2	5.3 ± 1.1	0.180
ANC (/µL)	2966.8 ± 846.0	2900.5 ± 862.3	0.878
Hemoglobin (g/dL)	13.6 ± 1.1	13.4 ± 1.1	0.217
MCV (fL)	93.9 ± 3.2	93.7 ± 3.3	0.787
MCH (pg)	32.2 ± 1.4	32.0 ± 1.3	0.029
MCHC (g/dL)	34.3 ± 0.8	34.2 ± 0.6	0.198
Platelet count (10^3^/µL)	245.1 ± 86.1	239.7 ± 82.4	0.112
BUN (mg/dL)	15.5 ± 3.3	18.0 ± 4.3	0.001
Creatinine (mg/dL)	0.7 ± 0.2	0.7 ± 0.1	0.471
Albumin (g/dL)	4.4 ± 0.3	4.3 ± 0.3	0.007
Total cholesterol (mg/dL)	190.6 ± 43.5	190.6 ± 49.3	0.675
AST (IU/L)	26.3 ± 6.2	27.6 ± 6.0	0.110
ALT (IU/L)	20.9 ± 5.4	22.9 ± 10.3	0.234
GGT (IU/L)	28.7 ± 35.4	30.0 ± 36.6	0.462
Total bilirubin (mg/dL)	0.7 ± 0.3	0.7 ± 0.2	0.497
CRP (mg/L)	0.8 ± 0.7	0.7 ± 0.6	0.533
**Underlying diseases**			
Hypertension	5 (14.3)		
Diabetes mellitus	5 (14.3)		

BMI, body mass index; WBC, white blood cell; ANC, absolute neutrophil count; MCV, mean corpuscular volume; MCH, mean corpuscular hemoglobin; MCHC, mean corpuscular hemoglobin concentration; BUN, blood urea nitrogen; AST, aspartate transaminase; ALT, alanine transaminase; GGT, gamma-glutamyl transferase; CRP, C-reactive protein; HBV, hepatitis B. Continuous variables are shown as median ± interquartile range (IQR) and categorical variables as numbers (percentage).

**Table 2:** Differential relative taxonomic abundance before and after booster vaccination (LDA Effect Size >2.5).

**Increased**					
**Taxon Name**	**Taxon Rank**	**B1**	**B2**	**LDA Effect Size**	***p* value**
*Verrucomicrobiae*	Class	0.05867	0.48008	3.35640	0.03572
*Coriobacteriia*	Class	0.30181	0.33425	2.91045	0.04755
*Verrucomicrobiales*	Order	0.05867	0.48008	3.32644	0.03572
*Coriobacteriales*	Order	0.30181	0.33425	2.91045	0.04755
*Akkermansiaceae*	Family	0.05867	0.48008	3.34064	0.03572
*Coriobacteriaceae*	Family	0.30181	0.33425	2.91045	0.04755
*Akkermansia*	Genus	0.05867	0.48008	3.34346	0.03572
*Collinsella*	Genus	0.07894	0.21245	2.75135	0.04464
*Anaerotruncus*	Genus	0.00388	0.02863	2.23196	0.01588
*Bacteroides fragilis*	Species	1.36713	2.93033	4.03647	0.02999
*Fusobacterium necrogenes*	Species	0.71276	1.39400	3.89480	0.02290
*Akkermansia muciniphila*	Species	0.05867	0.46066	3.31958	0.04992
*Ruminococcaceae PAC000661_g PAC001052_s*	Species	0.11072	0.19429	2.84822	0.02400
*Collinsella aerofaciens*	Species	0.07894	0.21245	2.75135	0.04464
*Oscillibacter KI271778_s*	Species	0.04889	0.14275	2.70632	0.04564
**Decreased**					
**Taxon name**	**Taxon Rank**	**B1**	**B2**	**LDA Effect Size**	***p* value**
*Bacteroidetes*	Phylum	50.18577	41.78608	4.63612	0.01297
*Bacteroidia*	Class	50.18534	41.78566	4.63612	0.01297
*Bacteroidales*	Order	50.18534	41.78566	4.63612	0.01297
*Lachnospiraceae_uc*	Genus	0.02531	0.01802	2.02576	0.00082
*Prevotella bivia*	Species	1.87355	0.28048	4.00505	0.03145

LDA, linear discriminant analysis.

**Table 3:** Species-level microbiota differences attributable to NVX-CoV2373 booster: a paired *t*-test analysis.

**Increased**				
**Taxon**	**B1 Average**	**B2 Average**	***t* value**	***p* value**
Bacteria; Bacteroidetes; Bacteroidia; Bacteroidales; Bacteroidaceae; Bacteroides; Bacteroides fragilis	524.44118	933.64706	−2.09804	0.04364
Bacteria; Firmicutes; Clostridia; Clostridiales; Lachnospiraceae; Blautia; Blautia glucerasea	0.00000	0.14706	−2.38530	0.02296
Bacteria; Firmicutes; Clostridia; Clostridiales; Christensenellaceae; PAC001360_g; FJ367045_s	0.97059	3.44118	−2.30547	0.02757
Bacteria; Firmicutes; Clostridia; Clostridiales; Ruminococcaceae; Pseudoflavonifractor; Flavonifractor plautii	43.23529	114.41176	−2.48175	0.01834
Bacteria; Firmicutes; Clostridia; Clostridiales; Ruminococcaceae; Oscillibacter; KI271778_s	18.47059	43.91176	−2.17134	0.03720
Bacteria; Firmicutes; Bacilli; Lactobacillales; Lactobacillaceae; Lactobacillus; Lactobacillus intestinalis	0.00000	0.11765	−2.09762	0.04368
Bacteria; Firmicutes; Bacilli; Lactobacillales; Lactobacillaceae; Lactobacillus; Lactobacillus murinus	0.00000	0.11765	−2.09762	0.04368
Bacteria; Firmicutes; Clostridia; Clostridiales; Ruminococcaceae; PAC001637_g; PAC001637_s	4.23529	8.08824	−2.17707	0.03673
Bacteria; Firmicutes; Clostridia; Clostridiales; Lachnospiraceae; PAC002152_g; PAC002152_s	0.02941	0.20588	−2.24364	0.03169
Bacteria; Firmicutes; Bacilli; Lactobacillales; Streptococcaceae; Streptococcus; Streptococcus salivarius	22.20588	42.94118	−2.21749	0.03359
**Decreased**				
**Taxon**	**B1 Average**	**B2 Average**	***t* value**	***p* value**
Bacteria; Firmicutes; Tissierellia; Tissierellales; Peptoniphilaceae; Anaerococcus; Anaerococcus lactolyticus	0.58824	0.08824	2.15322	0.03870
Bacteria; Actinobacteria; Coriobacteriia; Coriobacteriales; Coriobacteriaceae; Gordonibacter; Gordonibacter pamelaeae	0.11765	0.00000	2.09762	0.04368
Bacteria; Proteobacteria; Betaproteobacteria; Burkholderiales; Oxalobacteraceae; Oxalobacter; KI392030_s	1.67647	0.41176	2.10603	0.04289
Bacteria; Fusobacteria; Fusobacteria_c; Fusobacteriales; Leptotrichiaceae; Sneathia; Leptotrichia amnionii	2.44118	0.67647	2.11907	0.04170
Bacteria; Firmicutes; Clostridia; Clostridiales; Lachnospiraceae; Marvinbryantia; PAC002376_s	1.94118	0.82353	2.08135	0.04524
Bacteria; Bacteroidetes; Bacteroidia; Bacteroidales; Porphyromonadaceae; Parabacteroides; Parabacteroides_uc	24.64706	12.79412	2.12536	0.04113

**Table 4:** Taxonomic profile-based changes in functional abundance (LDA Effect Size >2.5).

**Increased**			
**Ortholog**	**Definition**	**LDA Effect Size**	***p* value**
-	-	-	-
**Module (PICRUSt)**	**Definition**	**LDA Effect Size**	***p* value**
M00207	Putative multiple sugar transport system	2.57795109	0.026388634
M00038	Tryptophan metabolism, tryptophan => kynurenine => 2-aminomuconate	2.539665479	0.047659036
**Module (MinPath)**	**Definition**	**LDA effect size**	***p* value**
-	-	-	-
**Pathway (PICRUSt)**	**Definition**	**LDA effect size**	***p* value**
-	-	-	-
**Pathway (MinPath)**	**Definition**	**LDA effect size**	***p* value**
-	-	-	-
**Decreased**			
**Ortholog**	**Definition**	**LDA effect size**	***p* value**
-	-	-	-
**Module (PICRUSt)**	**Definition**	**LDA effect size**	***p* value**
M00126	Tetrahydrofolate biosynthesis, GTP ≥ THF	2.60949489	0.022580649
**Module (MinPath)**	**Definition**	**LDA effect size**	***p* value**
-	-	-	-
**Pathway (PICRUSt)**	**Definition**	**LDA effect size**	***p* value**
ko01100	Metabolic pathways	3.204292376	0.033634627
**Pathway (MinPath)**	**Definition**	**LDA effect size**	***p* value**
ko04210	Apoptosis	2.855686688	0.005164992
ko04974	Protein digestion and absorption	2.748775013	0.040129783

LDA, linear discriminant analysis.

**Table 5:** Baseline characteristics and antibody responses: comparison between low and high responders to NVX-CoV2373 booster.

Characteristics	Low Responders (*n* = 27)	High Responders (*n* = 8)	*p* Value
**Age (years)**	65.1 ± 4.0	64.5 ± 4.8	0.527
**Female sex (%)**	8 (29.6)	4 (50.0)	0.402
**BMI (kg/m^2^)**	23.8 ± 2.7	23.4 ± 1.8	0.582
**Anti-SARS-CoV-2 S IgG (U/mL)**	15,213.8 ± 11,500.9	1792.5 ± 679.5	<0.001
**Laboratory test results**			
WBC (10^3^/µL)	5.6 ± 1.2	5.3 ± 1.2	0.783
ANC (/µL)	2961.4 ± 775.0	2985.2 ± 1116.0	0.773
Hemoglobin (g/dL)	13.5 ± 1.0	13.8 ± 1.4	0.651
MCV (fL)	93.1 ± 3.0	96.6 ± 2.4	0.006
MCH (pg)	31.8 ± 1.2	33.5 ± 1.5	0.015
MCHC (g/dL)	34.2 ± 0.6	34.7 ± 1.1	0.145
Platelet count (10^3^/uL)	244.8 ± 92.8	246.1 ± 63.5	0.922
BUN (mg/dL)	15.0 ± 3.4	17.2 ± 2.4	0.065
Creatinine (mg/dL)	0.7 ± 0.2	0.8 ± 0.2	0.568
Albumin (g/dL)	4.5 ± 0.2	4.3 ± 0.3	0.281
Total cholesterol (mg/dL)	195.3 ± 45.1	174.8 ± 35.5	0.179
AST (IU/L)	25.4 ± 3.3	29.2 ± 11.5	0.363
ALT (IU/L)	20.8 ± 5.4	21.2 ± 5.7	0.768
GGT (IU/L)	22.5 ± 12.8	49.8 ± 69.4	0.129
Total bilirubin (mg/dL)	0.7 ± 0.3	0.7 ± 0.3	>0.999
CRP (mg/L)	0.8 ± 0.8	0.8 ± 0.4	0.316
**Underlying diseases**			
Hypertension	3 (11.1)	2 (25.0)	0.568
Diabetes mellitus	4 (14.8)	1 (12.5)	>0.999
Dyslipidemia			
HBV carrier			

BMI, body mass index; WBC, white blood cell; ANC, absolute neutrophil count; MCV, mean corpuscular volume; MCH, mean corpuscular hemoglobin; MCHC, mean corpuscular hemoglobin concentration; BUN, blood urea nitrogen; AST, aspartate transaminase; ALT, alanine transaminase; GGT, gamma-glutamyl transferase; CRP, C-reactive protein; HBV, hepatitis B. Continuous variables are shown as median ± interquartile range (IQR) and categorical variables as numbers (percentage).

**Table 6:** Differential taxonomic abundance before NVX-CoV2373 booster vaccination: comparison between low and high responders (LDA Effect Size >2.5).

**Low Responders > High Responders**					
**Taxon Name**	**Taxon Rank**	**Low Responders**	**High Responders**	**LDA Effect Size**	***p* value**
*Mogibacterium_f*	Family	0.13663	0.03862	2.68501	0.01903
*Alloprevotella*	Genus	1.34650	0.00423	3.88420	0.00919
*Agathobacter*	Genus	1.22359	0.10317	3.68815	0.01125
*Paraprevotella*	Genus	0.27037	0.00053	3.10451	0.00408
*Agathobacter rectalis*	Species	1.22112	0.10317	3.68731	0.01125
*Oscillibacter PAC001129_s*	Species	0.60892	0.04868	3.43149	0.01566
*Paraprevotella clara*	Species	0.25281	0.00053	3.07496	0.00660
*PAC001052_s*	Species	0.14143	0.00000	2.85678	0.04837
*Oscillibacter_uc*	Species	0.10069	0.03545	2.58717	0.04359
**Low Responders < High Responders**					
**Taxon name**	**Taxon rank**	**Low Responders**	**High Responders**	**LDA effect size**	***p* value**
*Pasteurellales*	Order	0.13045	0.21111	2.76005	0.01753
*Pasteurellaceae*	Family	0.13045	0.21111	2.75910	0.01753
*Ruminococcus_g5*	Genus	0.28285	0.39735	3.21498	0.02741
*Haemophilus*	Genus	0.13045	0.20847	2.75605	0.01753
*Romboutsia*	Genus	0.05624	0.13862	2.65415	0.02366
*Agathobaculum*	Genus	0.12757	0.22116	2.63919	0.04995
*Faecalimonas*	Genus	0.00027	0.09841	2.62938	0.01406
*Ruminococcus gnavus*	Species	0.28285	0.39735	3.21498	0.02741
*Bacteroides PAC002300_s*	Species	0.00014	0.33016	3.17412	0.03772
*Bacteroides PAC002364_s*	Species	0.00069	0.30053	3.12731	0.00062
*Alistipes finegoldii*	Species	0.01084	0.18783	3.00748	0.01906
*Faecalimonas umbilicata*	Species	0.00027	0.09841	2.61101	0.01406
*Agathobaculum butyriciproducens*	Species	0.10082	0.17460	2.56622	0.04077
*Clostridium_g24 PAC001295_s*	Species	0.03429	0.10635	2.55174	0.01099

LDA, linear discriminant analysis.

**Table 7:** Differences in functional abundance based on taxonomic profiles before NVX-CoV2373 booster vaccination: comparison between low and high responders (LDA Effect Size >2.5).

**Low Responders > High Responders**			
**Ortholog**	**Definition**	**LDA effect size**	***p* value**
-	-	-	-
**Module (PICRUSt)**	**Definition**	**LDA effect size**	***p* value**
-	-	-	-
**Module (MinPath)**	**Definition**	**LDA effect size**	***p* value**
M00389	APC/C complex	2.54234332	0.01271625
**Pathway (PICRUSt)**	**Definition**	**LDA effect size**	***p* value**
-	-	-	-
**Pathway (MinPath)**	**Definition**	**LDA effect size**	***p* value**
-	-	-	-
**Low Responders < High Responders**			
**Ortholog**	**Definition**	**LDA effect size**	***p* value**
-	-	-	-
**Module (PICRUSt)**	**Definition**	**LDA effect size**	***p* value**
-	-	-	-
**Module (MinPath)**	**Definition**	**LDA effect size**	***p* value**
-	-	-	-
**Pathway (PICRUSt)**	**Definition**	**LDA effect size**	***p* value**
-	-	-	-
**Pathway (MinPath)**	**Definition**	**LDA effect size**	***p* value**
ko05014	Amyotrophic lateral sclerosis (ALS)	2.756572204	0.020273273

LDA, linear discriminant analysis.

## Data Availability

Data and materials supporting the findings of this study are available through the NCBI Sequence Read Archive (SRA). The dataset has been deposited under the BioProject accession number PRJNA1066510 (Temporary Submission ID: SUB15774697). The data will be publicly released on 30 June 2027, or upon publication, whichever occurs first. After the release date, the sequencing data will be accessible at: https://www.ncbi.nlm.nih.gov/sra/PRJNA1066510 (accessed on 8 December 2025).
